# Germline and somatic variant identification using BGISEQ-500 and HiSeq X Ten whole genome sequencing

**DOI:** 10.1371/journal.pone.0190264

**Published:** 2018-01-10

**Authors:** Ann-Marie Patch, Katia Nones, Stephen H. Kazakoff, Felicity Newell, Scott Wood, Conrad Leonard, Oliver Holmes, Qinying Xu, Venkateswar Addala, Jenette Creaney, Bruce W. Robinson, Shujin Fu, Chunyu Geng, Tong Li, Wenwei Zhang, Xinming Liang, Junhua Rao, Jiahao Wang, Mingyu Tian, Yonggang Zhao, Fei Teng, Honglan Gou, Bicheng Yang, Hui Jiang, Feng Mu, John V. Pearson, Nicola Waddell

**Affiliations:** 1 Department of Genetics and Computational Biology, QIMR Berghofer Medical Research Institute, Brisbane, Queensland, Australia; 2 National Centre for Asbestos Related Disease, School of Medicine and Pharmacology, University of Western Australia, Nedlands, Western Australia, Australia; 3 Department of Respiratory Medicine, Sir Charles Gairdner Hospital, Nedlands, Western Australia, Australia; 4 BGI, BGI-Shenzhen, Shenzhen, China; CNR, ITALY

## Abstract

Technological innovation and increased affordability have contributed to the widespread adoption of genome sequencing technologies in biomedical research. In particular large cancer research consortia have embraced next generation sequencing, and have used the technology to define the somatic mutation landscape of multiple cancer types. These studies have primarily utilised the Illumina HiSeq platforms. In this study we performed whole genome sequencing of three malignant pleural mesothelioma and matched normal samples using a new platform, the BGISEQ-500, and compared the results obtained with Illumina HiSeq X Ten. Germline and somatic, single nucleotide variants and small insertions or deletions were independently identified from data aligned human genome reference. The BGISEQ-500 and HiSeq X Ten platforms showed high concordance for germline calls with genotypes from SNP arrays (>99%). The germline and somatic single nucleotide variants identified in both sequencing platforms were highly concordant (86% and 72% respectively). These results indicate the potential applicability of the BGISEQ-500 platform for the identification of somatic and germline single nucleotide variants by whole genome sequencing. The BGISEQ-500 datasets described here represent the first publicly-available cancer genome sequencing performed using this platform.

## Introduction

The human genome project was an important achievement in life sciences and paved the way for major technology developments in DNA sequencing. The development of next generation sequencing (NGS, also known as massively parallel or high-throughput sequencing) machines commenced with the 454 DNA sequencer (Life Sciences), followed by the Genome Analyzer (Solexa) and SOLiD (Agencourt) platforms. Solexa, who pioneered sequencing by synthesis technology, were acquired by Illumina who further refined the technology and developed the HiSeq sequencers (reviewed in [1, 2]). The HiSeq platforms have now produced the majority of the publicly available human DNA sequencing data. Over time the cost of sequencing has decreased and the technology has become more accessible, both in terms of sequence hardware and tools for analysis, which has resulted in NGS being adopted by many researchers.

NGS has been applied in cancer research to identify somatic mutations occurring in many tumour types. Two large consortia, The Cancer Genome Atlas (TCGA) [3] and the International Cancer Genome Consortium (ICGC) [4], have sequenced thousands of tumours from over 50 cancer types. These two consortia have been instrumental in increasing our knowledge of cancer genomics and have identified significantly mutated genes, candidate actionable mutations and mutational processes [5] that occur during tumour development.

To date most large scale cancer genome studies have utilised the Illumina HiSeq platforms. In 2015, Beijing Genomics Institute (BGI) launched the BGISEQ-500 as alternative to existing short-read sequencing technologies. The BGISEQ-500 is based on combinatorial Probe-Anchor Synthesis and improved DNA Nanoballs technology [6]. Previously the BGISEQ-500 has been used to sequence small non-coding RNAs [7], insect derived transcriptomes [8], genomes from historic and ancient dog and wolf samples [9] and the whole genome of a single human DNA reference sample [10]. However to date no studies have used the platform for cancer whole genome sequencing (WGS). Here we evaluate WGS data generated on the BGISEQ-500 and HiSeq X Ten using DNA extracted from cancer and matched germline samples from patients with malignant pleural mesothelioma.

## Materials and methods

### Patients and samples

Samples were collected from three patients (identified as 9869, 11202 and 11398) diagnosed with malignant pleural mesothelioma at the Sir Charles Gairdner Hospital in Perth, Western Australia. The work in this study was approved by the Human Research Ethics Committee of Sir Charles Gairdner Hospital and QIMR Berghofer Medical Research Institute and all patients provided written consent. Blood samples were collected in K_2_EDTA plasma Vacutainer tubes (BD Bioscience, New Jersey, USA). Pleural effusion samples were collected without preservative by routine pleurocentesis and were in excess to that required for diagnosis. A diagnosis of malignant pleural mesothelioma was confirmed by pathologists experienced in the diagnosis of effusions. Effusions were centrifuged for 10 min at 1000 g and the resulting cell pellet was washed in PBS by centrifugation at 400 g for 10 min then depleted of CD45 positive cells using the EasyStep Human CD45 Depletion kit (Stemcell technologies, Vancouver, Canada). Resulting cellular composition was reviewed on cytospin cell preparations.

### DNA extraction and quality assessment

DNA was extracted using the AllPrep DNA/RNA/miRNA Universal kit (Qiagen) following the manufacturer’s instructions. DNA samples extracted from blood and matched pleural effusion samples were quantified using a Qubit (ThermoFisher Scientific). To ensure that there was high tumour content in each sample the DNA was assayed using SNP arrays (Infinium Omni2.5–8, Illumina) and tumour content estimated using qPure [11]. The tumour content was 89% for patient 9869; 78% for patient 11202 and 81% for patient 11398. A total of 2 μg of each DNA sample was sent to both BGI and the Kinghorn Centre for Clinical Genomics (KCCG) for WGS using the BGISEQ-500 and HiSeq X Ten, respectively.

### Library construction and whole genome sequencing

Sequence libraries for the BGISEQ-500 platform were prepared using a sonication or fragmentase based library construction method. The MGIEasy^™^ DNA Library Prep Kit V1 (BGI, Cat. No. 85-05533-00) was applied to construct the sonication based library using 1000ng of genomic DNA that had been sheared with an E220 Covaris instrument (Covaris Inc.) following the manufacturer’s manual. The fragmentase based WGS libraries used 100ng of each genomic DNA sample that was sheared by fragmentase (NEB). All samples described were prepared using the fragmentase-based library method, except for the normal samples from 9869 which underwent sonication. After fragmentation by sonication or fragmentase, the DNA fragments were size selected using AMpure XP Beads (Beckman Coulter, Indiana, USA) and then underwent end-repairing, phosphorylation and A-tailing reactions. BGISEQ-500 platform-specific adaptors were ligated to the A-tailed fragments, and the ligated fragments were purified, and then amplified using PCR. Finally, circularization was performed to generate single stranded DNA circles. After quantitation and qualification, the libraries were sequenced.

BGI performed the DNA nanoball preparation and whole genome sequencing using the circular single stranded libraries as a template for rolling circle amplification to form DNA nanoballs. The DNA nanoballs were loaded onto a sequencing flow cell and then processed for 50 bp paired-end sequencing on the BGISEQ-500 platform. In contrast the KCCG performed WGS on a HiSeq X Ten using the HiSeq X Ten Reagent Kit v2.5 following manufacturer’s guidelines.

### Whole genome sequence analysis

Whole genome sequencing was performed as 50 bp paired end using the BGISEQ-500 platform and 150 bp paired end on the HiSeq X Ten. The BGISEQ-500 sequence data has been deposited into the EGA (Accession number: EGAS00001002298) and the Illumina data is available in the EGA (Accession number: EGAS00001002299). Data from the BGISEQ-500 and HiSeq X Ten was analysed using the same pipeline. Essentially, sequence reads were trimmed using Cutadapt (version 1.11), aligned to GRCh37 using BWA-MEM (version 0.7.12-r1039), duplicates marked with Picard (version 1.129, http://picard.sourceforge.net) and coordinates sorted using Samtools (version 1.3) [12]. Single nucleotide substitution variants (SNV) were detected using a dual calling strategy using qSNP [13] and GATK HaplotypeCaller [14]. Short insertion and deletions (indels) of ≤50bp, were also called with the GATK Haplotype caller. Variants were annotated with Ensembl v75 gene feature information and transcript or protein consequences using SnpEff (version 4.2) [15]. All germline SNV and indels were annotated with whether they are present in the genome Aggregation Database (gnomAD), which is comprised of two datasets: exome sequence data from the Exome Aggregation Consortium[16] and whole genome sequencing from 15,496 individuals. Variants were considered “called” and used in subsequent analysis if they passed the following filters: a minimum read depth of 8 reads in the normal control data and 12 in the tumour data; at least 4 reads containing the variant where the variant was identified on both strands and not within the first or last 5 bases. Additionally, indels that were located immediately adjacent to homopolymer regions of at least 6 bp and for which the inserted or deleted base were identical to the homopolymer base were filtered. Variants that did not pass these filters were considered “low evidence”. The processes used to analyse the somatic data were established for the International Cancer Genome Consortia (ICGC)[4] and have been used for several high impact cancer studies[17–19]. These processes have also been internationally benchmarked against other pipelines [20]. In this manuscript the term ‘somatic variants’ refers to mutations acquired by the tumour, or tumour specific variants which are not present in the germline (matched normal sample).

### Comparison of variants detected between different platforms

The germline genotypes from the SNP arrays were compared to the BGISEQ-500 and HiSeq X Ten sequence data where sequencing read depth required ≥10 reads. This resulted in 525,029 and 521,040 SNPs from the SNP array being compared with the BGI and Illumina sequence data respectively for patient 9869; 504,234 and 504,352 SNPs for patient 11202; and 503,527 and 512,213 SNPs for patient 11398.

The chromosome position and genotype of each germline and somatic variant called from each sequence platform was used to compare and identify the SNVs and indels which were only detected in either the BGISEQ-500 or HiSeq X Ten datasets. A sequence pileup to count the bases present at each discordant position was performed to reveal any evidence of the variant at each locus Quality filtering was also employed during the pileup analysis to ensure only non-duplicate marked reads that contained a minimum of 35 matched bases as reported in the CIGAR string and 3 or fewer mismatches in the sequencing MD field were counted.

## Results

### Whole genome sequencing coverage

**Fig 1:**
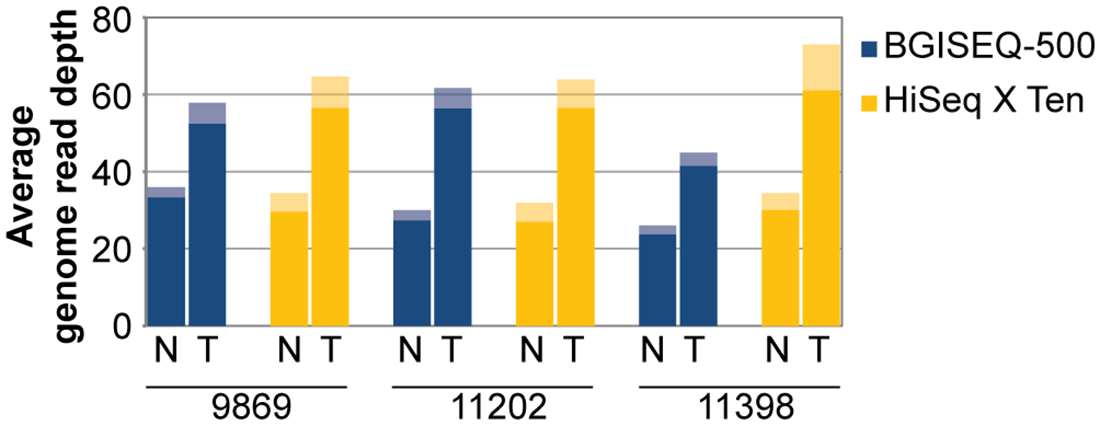
Average genome read depth using BGISEQ-500 and HiSeq X Ten data. The average whole-genome sequencing read depth for each platform (blue BGISEQ-500, yellow HiSeq X Ten), for each tumour (T) and normal (N) sample is displayed for three mesothelioma patients (9869, 11202 and 11398). Prior to variant calling sequence reads underwent quality filtering, and the subsequent average read depth remained similar between sequencing platforms, this is a more relevant measure of read depth as it represents the ‘usable’ portion of the sequencing data for detecting variants. The average quality-filtered sequencing read depth is indicated by the shaded bar.

The average non-duplicate sequencing read depth achieved by the BGISEQ-500 (50 base pair read length) and HiSeq X Ten (150 base pair read length) platforms was similar both before and after filtering by alignment quality (Fig 1). In the BGISEQ-500 data the average post-quality filtering read depth was 28X (range 24-33X) in the normal and 50X (range 41-56X) in the tumour samples and in the HiSeq X Ten data 29X (range 27-30X) in the normal and 58X (range 57-61X) in the tumour samples.

### Germline SNV and indel variant detected by each platform

The sequence data generated on the BGISEQ-500 and the HiSeq X Ten platforms showed a >99% concordance with the genotypes obtained from the Illumina SNP arrays (Table 1), indicating that both platforms were able to accurately detect common germline SNV assayed by the SNP arrays.

**Table 1:** The percent concordance of germline genotypes ascertained by SNP arrays compared to the BGISEQ-500 and HiSeq X Ten data.

Patient	SNP array vs BGISEQ-500	SNP array vs HiSeq X Ten
**9869**	99.797	99.789
**11202**	99.794	99.794
**11398**	99.797	99.795

**Fig 2:**
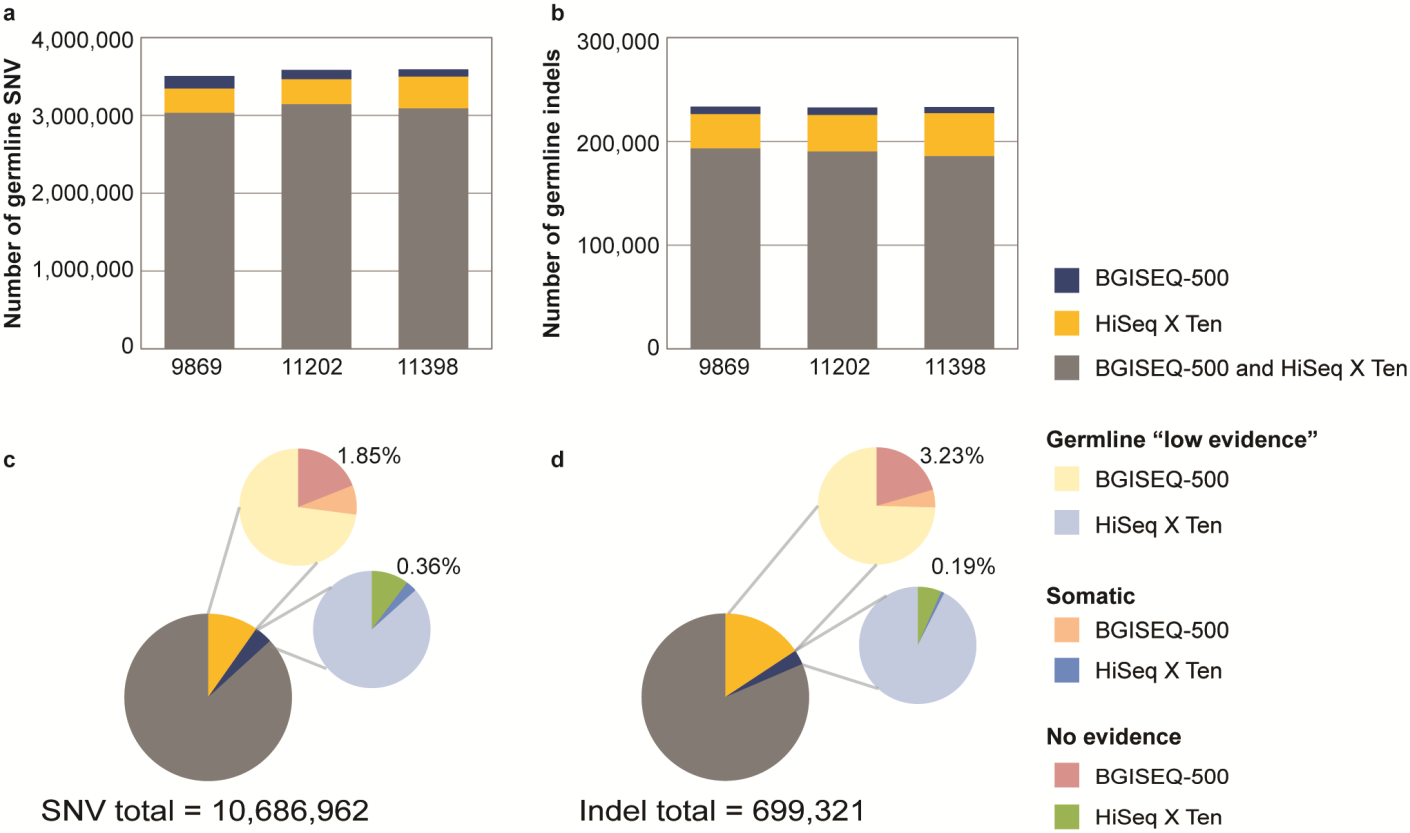
Germline variants identified in three mesothelioma samples (patients: 9869, 11202 and 11398) using BGISEQ-500 and HiSeq X Ten data. The number of germline SNV (**a**) and indels (**b**) identified in each patient using the BGISEQ-500 and HiSeq X Ten platforms. We investigated germline SNV (**c**) and indels (**d**) which were only called in one platform and that fall into three categories: i) identified as germline in the other platform but with low evidence; ii) identified in the other platform but predicted as a somatic variant; or iii) not identified in the other platform. Across the 3 patients only 197,434 (1.85%) SNVs were truly unique to the HiSeq X Ten and not identified in the BGISEQ-500 (c). Similarly in the BGISEQ-500 platform only 38,236 SNVs (0.36% of the total) were truly unique to the BGISEQ-500, not called in the HiSeq X Ten data (c). The same pattern was observed for indels (d), only 3.23% were unique to HiSeq X Ten and 0.19% to BGISEQ-500.

A summary of the number of germline and somatic SNV and indels identified with the BGISEQ-500 and HiSeq X Ten sequencing platforms is provided in Table 2. Across the genome the BGISEQ-500 and HiSeq X Ten platforms called an average of 3,562,321 germline SNV in each patient (representing 3,508,123; 3,586,280; and 3,592,559 germline SNV in patients: 9869, 11202 and 11398 respectively). The majority of the germline SNV (86%) were identified in both sequencing platforms (Fig 2a). However, across the 3 patients there were a total of 1,042,608 SNV which were only called by the HiSeq X Ten analyses and comprised 8.9%, 9.0% and 11.4% of the SNV identified in the 3 patient samples (patients: 9869, 11202 and 11398 respectively). There were less calls unique to BGISEQ-500 (371,514 SNV) which represented 4.6%, 3.3% and 2.6% of the SNV in the 3 patient samples (patients: 9869, 11202 and 11398 respectively). An average of 232,987 germline indels were called in each patient (representing 233,527; 232,260 and 233,174 germline indels in patients: 9869, 11202 and 11398 respectively) (Fig 2b). The majority of these indels (81.5%) were identified by both of the sequencing platforms, with only 15.7% called in the HiSeq X Ten only (representing 109,876 indels) and 2.8% (19,745 indels) called in the BGISEQ-500 data.

**Table 2:** Number of germline and somatic variants identified in three mesothelioma samples using whole genome sequencing. The percentage of the germline variants identified in this study and reported in European population data from gnomAD are presented in brackets.

		SNV				Indels			
		9869	11202	11398	All Patients	9869	11202	11398	All Patients
Germline	Identified in both platforms	3,033,980	3,146,317	3,092,543	9,272,840	193,359	190,436	185,905	569,700
		(96.8%)	(96.8%)	(96.8%)	(96.8%)	(91.7%)	(91.8%)	(92%)	(91.8%)
	HiSeq X Ten only	313,015	321,627	407,966	1,042,608	33,143	35,253	41,480	109,876
		(42.3%)	(42.3%)	(41.9%)	(42.1%)	(58.5%)	(58.4%)	(59.2%)	(58.7%)
	BGISEQ-500 only	161,128	118,336	92,050	371,514	7,025	6,931	5,789	19,745
		(4%)	(2.4%)	(4.1%)	(3.55%)	(13.8%)	(13.8%)	(11.6%)	(13.1%)
	Total	3,508,123	3,586,280	3,592,559	10,686,962	233,527	232,620	233,174	699,321
Somatic	Identified in both platforms	3,554	2,342	1,955	7,851	197	168	114	479
	HiSeq X Ten only	697	424	411	1,532	135	93	78	306
	BGISEQ-500 only	540	474	493	1,507	102	156	229	487
	Total	4,791	3,240	2,859	10,890	434	417	421	1,272

### Discordant germline SNV and indels between the different sequencing platforms

A proportion of SNVs and indels that were called germline in only one platform were either: i) identified as low evidence germline in the other platform; ii) identified in the other platform but predicted as a somatic variant; or iii) not identified in the other platform (Table 2, Fig 2c and 2d). Of the 10,686,962 SNVs called across the 3 data sets, 1,042,608 (9.76%) SNV that were called germline in the HiSeq X Ten platform only, 7.1% (760,482) were identified as low evidence in the BGISEQ-500 data; 0.79% (84,692) were identified in the BGISEQ-500 data but predicted as somatic which suggests that the alternate allele was not sequenced in the normal due to low coverage or sampling; only a small percentage of the total SNVs 1.85% (197,434) were uniquely identified in the HiSeq X Ten (Fig 2c). The same pattern was observed for BGISEQ-500, 3.48% of the SNV were called only in this platform, with 3.01% (321,937) identified as germline low evidence in the HiSeq X Ten data; 0.11% (11,341) were predicted as somatic in the HiSeq X Ten data; and only 0.36% (38,236) were uniquely identified in the BGISEQ-500 (Fig 2c). Similar to the SNV calls, the majority of discordant indel variants were actually detected but as low evidence in the other platform (Fig 2d). Of the total 699,321 indels identified 15.71% (109,876) were identified in HiSeq X Ten platform only. When compared to low evidence calls 11.72, 75% (81,935) were also identified as low evidence germline in the BGISEQ-500 data; 0.77% (5,364) were identified as somatic in the BGISEQ-500 data; and 3.23% (22,577) remained uniquely identified in the HiSeq X Ten (Fig 2d). Similarly, of the 19,745 indels that were called only using the BGISEQ-500 platform 92% (18,268) were identified in the HiSeq X Ten data but as low evidence; 0.03% (175) were identified as somatic in the HiSeq X Ten data; and 0.19% (1,302) were uniquely identified in the BGISEQ-500 (Fig 2d).

**Fig 3:**
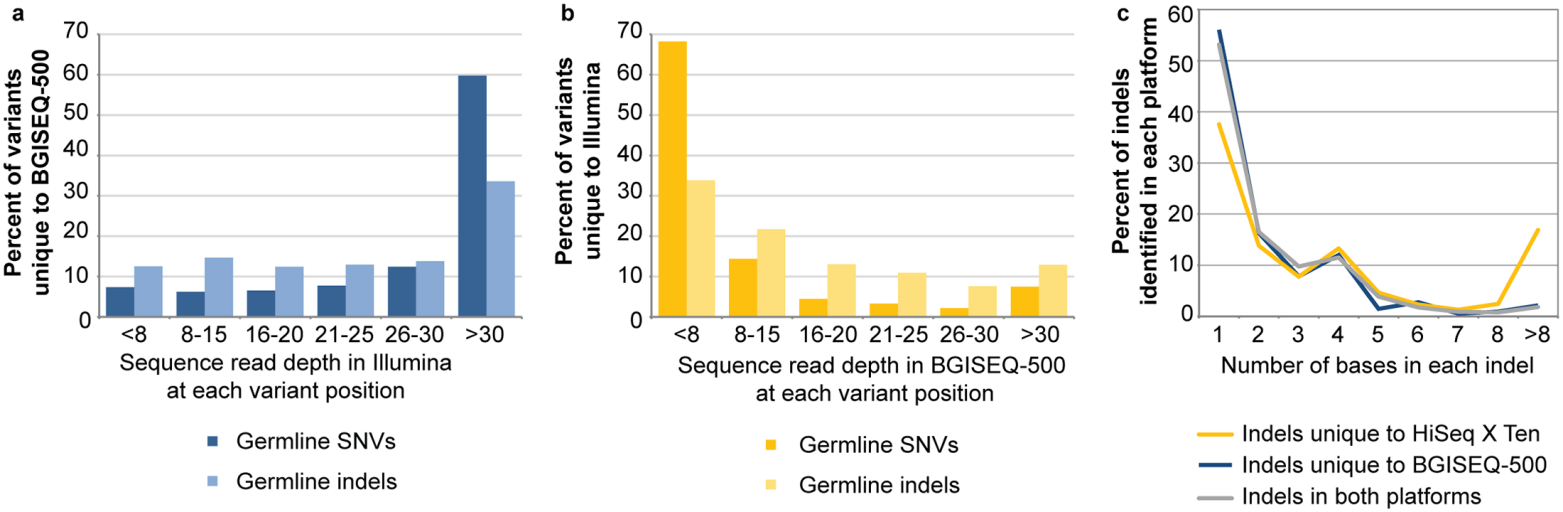
The sequence coverage of germline variants and the length of the indels which were identified in one sequence platform. Read depth in Illumina for variants unique to BGISEQ-500 (**a**) read depth in BGI for variants unique to Illumina (**b**). The distribution of the length (number of bases) of the indels that were identified in both sequencing platforms or unique to the HiSeq X Ten or BGISEQ-500 data is plotted (**c**).

To determine why a small proportion of the total germline calls across all patients were unique to each platform (0.36 and 1.85% SNV and 0.19 to 3.23% indels in the BGISEQ-500 and HiSeq X Ten respectively), an analysis of the read depth at the position of each variant was performed. Variants unique to the BGISEQ-500 data (38,236 SNV and 1,302 indels) were generally covered at a reasonable depth in the HiSeq X Ten data but no evidence for the variant was detected (Fig 3a). Such variants may not have been seen in the HiSeq X Ten data due to biases in the sampling of the variant allele. Alternatively, mapping errors affecting the shorter reads in the BGISEQ-500 may have led to artefact calls in regions that are difficult to map but were removed from the HiSeq X Ten data due to the >3 mismatches filter. Overall these variants, which are unique to the BGISEQ-500, represent a small number of the total germline SNV (38,236 of 10,686,962 or 0.36%) and indels (1,302 of 699,321 or 0.19%) identified from that platform. In contrast the majority (68%) of the 197,434 SNVs and 33% of the 22,577 indels that were unique to the HiSeq X Ten and not identified using the BGISEQ-500 were due to low sequence coverage across the variants positions (<8 reads in the normal) (Fig 3b). This may be due to random sampling during sequencing or that these regions in the genome are more problematic to sequence using the 50 bp paired end read lengths in the BGISEQ-500 data.

As an *in-silico* validation of germline calls we used the genome Aggregation Database (gnomAD) [16] to determine the occurrence of variants in the general population. The percentages of germline SNVs and indels present in the European population in gnomAD are included in Table 2. A total of 96.8% of the 9,272,840 SNVs called by both platforms have been reported in gnomAD. As expected the private variants in each platform have a much smaller representation in gnomAD. However, these variants are a small fraction of the total germline calls (3.4 and 9.76% of SNVs and 2.8 and 15% of indels for BGISEQ-500 and HiSeq XTen, respectively).

The size of the indels which were identified only in the HiSeq X Ten or BGISEQ-500 platform differed. The frequency of indels detected that were between 1–8 bps in length was similar between the platforms but the HiSeq X Ten data was able to detect a higher number of indels >8bp long (Fig 3c). This may be due the longer read length (150bp paired end) used in the HiSeq X Ten, as opposed to the 50 bp with the BGISEQ-500, as the longer read length will be able to align across larger indels more effectively. However a local realignment methodology may aid detection of longer indels in the shorter reads.

### Somatic SNV and indel variants detected by the different platforms

**Fig 4:**
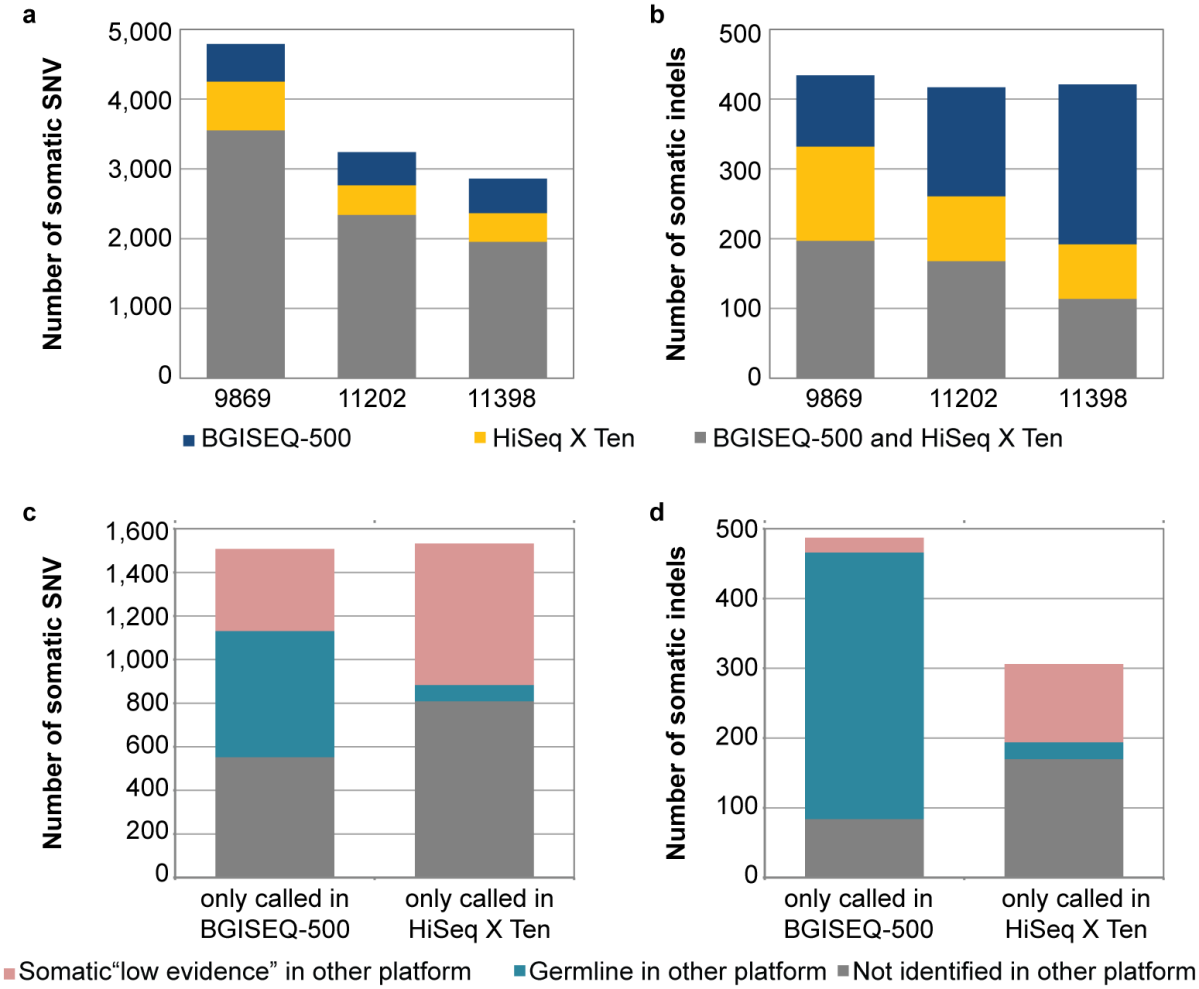
Somatic variants in mesothelioma patients identified using BGISEQ-500 and HiSeq X Ten data. A summary of the somatic variants identified in 3 mesothelioma patient samples (patient ID: 9869, 11202 and 11398) using different sequencing platforms. The number of somatic SNV (**a**) and indels (**b**) identified using the BGISEQ-500 and HiSeq X Ten platforms in each patient. The somatic SNV (**c**) and indels (**d**) which were only called in one platform fall into three categories: i) identified as somatic in the other platform but with low evidence; ii) identified in the other platform but predicted as a germline variant; or iii) not identified in the other platform.

A total of 10,890 somatic SNV were called using the HiSeq X Ten and BGISEQ-500 platforms across all three patients (representing 4,791; 3,240 and 2,859 somatic SNV in patients: 9869, 11202 and 11398 respectively). The majority of the somatic SNV (72%) were identified in both sequencing platforms, while 14% of the somatic SNVs were only called in the HiSeq X Ten data and 14% only called in the BGISEQ-500 data (Fig 4a). An average of 424 somatic indels were called using the HiSeq X Ten and BGISEQ-500 platforms each patient (representing 434; 417 and 421 somatic indels in patients: 9869, 11202 and 11398 respectively) (Fig 4b). Interestingly only 38% of the indels were identified by both sequencing platforms, while 14% were only called in the HiSeq X Ten and 38% only called in the BGISEQ-500. The high proportion of discordant somatic indel calls is not completely unexpected, as previous benchmarking studies have also found a higher discordant rate in somatic indels compared to SNV analysis[20]. In total 156 of the somatic mutations (141 SNV and 15 indels) were located in gene coding regions. Of these, 109 coding mutations (70%) were identified in both sequencing platforms and included the known mesothelioma driver gene, *BAP1* [21], while 20 mutations (13%) and 27 mutations (17%) were only called in the BGISEQ-500 and HiSeq X Ten data respectively (S1 Fig).

### Discordant somatic SNV and indel variants between the different platforms

Similar to the germline analysis the somatic SNV and indel variants which were called in one platform fell into three categories: i) identified as somatic in the other platform but as low evidence; ii) identified in the other platform but predicted as a germline variant; or iii) not identified in the other platform (Fig 4c and 4d). However compared to the germline calls, there were a higher proportion of SNV and indel variants which were unique to each platform. Also the somatic SNV and indels called in the BGISEQ-500 data contained a higher proportion of events which were identified as germline in the HiSeq X Ten platform (Fig 4c and 4d), which is likely due to biases against the variant allele in the normal sequencing data from the BGISEQ-500.

## Discussion

We sequenced three cancer and matched normal DNA pairs from mesothelioma patients using the BGISEQ-500 and HiSeq X Ten sequencing platforms. A comparison of the germline and somatic SNVs and indels detected using the BGISEQ-500 to those identified using the HiSeq X Ten platform revealed that the majority of variants were identified by both sequencing platforms. The three mesothelioma genomes are typical of that disease. They have a range of somatic mutations per megabase of between 0.85–1.52 which is at the low end of the spectrum of mutation load across many different cancers [22].

The small proportion of variants called in one platform but not the other are due to a multiplicity of factors. One key factor contributing to differences between the platform variant calls is the difference in read length between the two platforms (50 bp in the BGISEQ-500 and 150 bp in the HiSeq X Ten). Read length affects the ability to call variants primarily through alignment bias and error which are higher for short reads as there are fewer bases with which to uniquely align that read to the reference sequence. The effects of alignment bias are not evenly represented across the genome but are higher in AT-rich regions associated with repetitive, typically non-coding DNA. High concordance of known polymorphic SNP positions assessed by both the sequencing and array platforms are consistent with the selection of robust marker polymorphisms located within unique sequence regions. This suggests that alignment biases are much reduced in these selected sites. Read alignment was carried out using BWA-MEM, which is a development of the original Burrows-Wheeler Aligner algorithm, specifically designed for read lengths of over 70bp. It is reported that BWA-backtrack may perform better for reads shorter than 70bp. Alignment of the shorter BGI reads may have been penalised by BWA-MEM.

A further factor that may have contributed to the small discordance observed was the application of the same variant calling and analysis pipeline to both datasets. This pipeline was designed for use with long Illumina reads and may have penalised the analysis of the BGISEQ-500 data by requiring a minimum of 35 contiguous matched bases, and fewer than three mismatched bases within a read. This filtering step only removes reads failing these tests prior to variant calling with qSNP and it is not applied before processing with GATK Haplotype Caller. This means short BGISEQ-500 reads with hard or soft clipping of >16 bases or those containing indels would not contribute to variant detection using qSNP. The second part of the filter requires less than 3 mismatches and is much more likely to penalise the longer Illumina reads. This would leave short, poorly aligned BGISEQ-500 reads in regions prone to high alignment bias that could contribute to low quality variant calls.

To minimise the possibility of differences in the sample quality causing discordance we supplied an aliquot of high molecular weight DNA from the same nucleic acid extraction for all three sample pairs to each of the sequencing centres. Random sampling of DNA molecules during the library preparation and sequencing process are likely sources of discordant calls in our data. This source of error was evident in the germline calls detected in the data from only one platform but as a somatic call or as a low evidence call in the other. The failure to pass calling thresholds in just one of the platforms for a true positive variant is most likely due to this sampling affect. Library preparation for both platforms was different including the fragmentation processes, template size selection and cluster or DNA nanoball generation. These differences will introduce a degree of bias that could particularly affect somatic variant calling where the tumour specific signal may be reduced as compared with the germline signal. These platform specific differences would likely persist in any comparison. Use of a bespoke analysis pipeline, which better considers the shorter read lengths for BGISEQ-500 data may reduce some discordant calls but could also lead to a different set of discordant calls

Overall, the BGISEQ-500 and HiSeq X Ten sequencing platforms show a high concordance to germline genotypes ascertained from SNP arrays. Both sequencing platforms show a high concordance to each other in their ability to detect germline and somatic SNVs and indels.

## Supporting information

S1 Fig Protein coding mutations detected using BGISEQ-500 and HiSeq X Ten data. A summary of the genes affected by the protein coding mutations which were identified in 3 mesothelioma samples (patient ID: 9869, 11202 and 11398).
(TIF)
