# Supplementary figures and images for: Germline and somatic variant identification using BGISEQ-500 and HiSeq X Ten whole genome sequencing
Source: PLoS One. 2018 Jan 10;13(1):e0190264. doi: 10.1371/journal.pone.0190264 (PMC5761881; doi:10.1371/journal.pone.0190264)

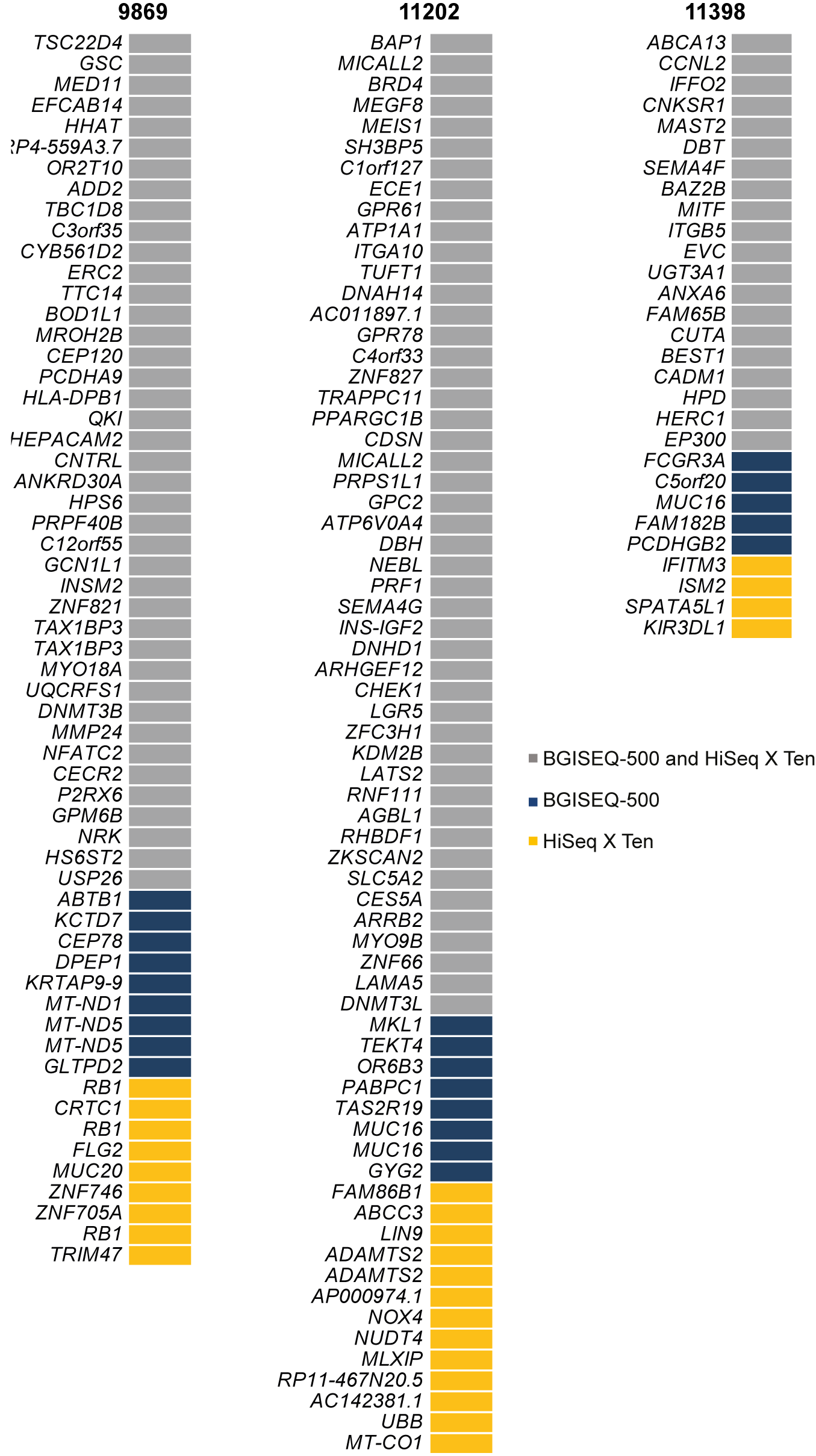

Supplement: S1 Fig — A summary of the genes affected by the protein coding mutations which were identified in 3 mesothelioma samples (patient ID: 9869, 11202 and 11398). (TIF) [file pone.0190264.s001.tif]
